# Neither the number of feature dimensions nor their task relevance necessarily affect interocular grouping in binocular rivalry

**DOI:** 10.3758/s13414-026-03265-0

**Published:** 2026-05-06

**Authors:** Marek A. Pedziwiatr, Monika Derda, Weronika Bator, Michał Wierzchoń, Christoph Teufel

**Affiliations:** 1 Centre for Brain Research, Jagiellonian University, Kopernika Street 50, 31-501 Kraków, Poland; 2 Institute of Social Science, University of Applied Sciences in Nowy Targ, Nowy Targ, Poland; 3 Consciousness Lab, Institute of Psychology, Jagiellonian University, Kraków, Poland; 4 Biological and Computational Vision Lab, Cardiff University Brain Research Imaging Centre, School of Psychology, Cardiff University, Cardiff, UK

**Keywords:** Binocular vision: Rivalry/Bistable Perception, Motion: *Other, 2D shape and form

## Abstract

When one eye views half of stimulus A and half of stimulus B, and the other eye views the respective other halves, perception fluctuates. Among percepts reflecting monocular inputs (indicating typical binocular rivalry), percepts of undivided stimuli A and B occur. They indicate the grouping of parts of inputs from both eyes together, a process called “interocular grouping”. Our study investigated if, similar to binocular rivalry, this process is affected by attentional modulation stemming from manipulating the task relevance of the stimulus features driving it. We used stimuli in which form (object identity), motion (motion direction), or both could drive interocular grouping. Contrary to our hypothesis, the grouping was not affected by designating either of these dimensions as task relevant. Moreover, unlike in previous studies, we did not observe stronger grouping when two features, instead of one, drove it. However, we found that our manipulation of task relevance influenced observers’ eye movements, which, in turn, was related to the number of perceptual changes the observers reported. We interpret this pattern of results as suggesting that the dynamics of perceptual fluctuations differed depending on the task. Overall, we show that the factors influencing interocular grouping strength are more complex than previously thought and not necessarily aligned with the factors influencing binocular rivalry. The data from this study can be accessed via the following link: 10.5281/zenodo.18924437.

**Supplementary Information:**

The online version contains supplementary material available at 10.3758/s13414-026-03265-0.

## Introduction

In most viewing situations, the inputs to the two eyes are similar and are typically fused into a stable, unitary percept. High dissimilarity of the two inputs, however, results in a phenomenon called binocular rivalry (Arnold, 2011a, b; O’Shea, 2011). During binocular rivalry, the input to one of the two eyes is perceived, while the input to the other eye is suppressed, and therefore not perceived. After a brief period during which a mosaic composed of fragments of both inputs is usually experienced (a “mixed percept”), the previously suppressed input gains dominance. In essence, in typical binocular rivalry, the observer’s experience keeps fluctuating between percepts of the two monocular inputs despite identical visual stimulation. Intriguingly, certain stimuli can also elicit fluctuations between percepts consisting of parts of inputs from *different* eyes (see Table 1 for an illustration). This phenomenon is called “interocular grouping” (IOG; Kovács et al., 1996; Papathomas et al., 2004). A well-known demonstration of IOG, devised by Diaz-Caneja (1928; translated into English in Alais et al., 2000; see also Ngo et al., 2000), uses an image, the left half of which contains red horizontal lines and the right half contains green concentric semi-circles. This image is presented to one of the observer’s eyes. The same image is presented to the other eye, but it is flipped along its vertical meridian. In this situation, observers experience fluctuations not only between percepts of both stimuli but – crucially – also between percepts consisting exclusively of stripes or circles. Therefore, although the Diaz-Caneja stimuli provide monocular inputs composed of both stripes and circles, the visual system binds similar stimulus parts together across the two eyes, which results in percepts of only stripes or circles.

**Table 1:** Illustration of stimuli, experimental instructions, and button-press reports indicating experiencing interocular grouping (IOG) percepts.

Stimuli left eye/right eye	Grouping cue	Instruction	Response that indicated IOG percepts
$\overrightarrow{A} \overrightarrow{B}/\overleftarrow{B} \overleftarrow{A}$	Form (F)	Same Form	Same
		Same Motion	Different
$\overrightarrow{A} \overleftarrow{A}/\overleftarrow{B} \overrightarrow{B}$	Motion (M)	Same Form	Different
		Same Motion	Same
$\overrightarrow{A} \overleftarrow{B}/\overleftarrow{B} \overrightarrow{A}$	Form and Motion (FM)	Same Form	Same
		Same Motion	Same
$\overrightarrow{A} \overrightarrow{A}/\overleftarrow{B} \overleftarrow{B}$	Null	Same Form	–
		Same Motion	–

Since Diaz-Caneja’s demonstration, studies using approaches ranging from neuroimaging (Buckthought et al., 2021) and Bayesian modelling (Jacot-Guillarmod et al., 2017) to pupillometry (Acquafredda et al., 2022) and other psychophysiological methods (Ngo et al., 2007) have provided insights into various aspects of IOG. In particular, the relationship between the stimulus properties, and the occurrence and strength of IOG has been well described. One of the key insights from this work is that the strength of IOG is primarily determined by the number of features driving it (Papathomas et al., 2004; see also Holten et al., 2016; Stuit et al., 2011; Van Lier & De Weert, 2003; Vergeer & Van Lier, 2010). For example, in Diaz-Caneja’s demonstration, two visual features driving the grouping (shape *and* color) lead to a more robust effect compared to a situation in which only one of them drives it (shape *or* color). In contrast, IOG strength is similar when stimulus parts that can be interocularly grouped generate percepts that have a coherent high-level interpretation (such as percepts of faces or optic flow patterns) and when they do not have such an interpretation (Holten et al., 2016; Stuit et al., 2014; see also Lee & Blake, 2004, and Mokri et al., 2023). This finding suggests that there are no or few influences of high-level interpretations on IOG. Another conclusion from these studies is that monocular information (i.e., information about the eye to which a certain stimulus part was presented), which, by necessity, operates “against” any factors that might drive IOG, is a strong driver of the integration of percepts across the visual field.

In contrast to stimulus properties, the influence of task-related attentional factors on IOG is not well understood, despite the fact that such influences are well established for binocular rivalry (Chong et al., 2005; Chopin & Mamassian, 2010; Einhäuser et al., 2021; Gayet et al., 2015; Lack, 1978; Meng & Tong, 2004; Mitchell et al., 2004; Ooi & He, 1999; Van Ee et al., 2005; Zhou & Hou, 2025; for reviews, see Dieter & Tadin, 2011; Paffen & Alais, 2011). For example, in a study investigating typical binocular rivalry, Gayet and colleagues (2015) instructed observers to memorize a color hue for a future memory test, rendering this feature task relevant. Before the memory test, observers experienced binocular rivalry between two colored gratings, out of which one had a color belonging to the same color category as the memorized hue. Results showed that during rivalry, this grating dominated for longer than the other one, indicating a “strengthening” effect of task relevance.

Binocular rivalry and IOG are thought to share common neural mechanisms (Tong et al., 2006). In the current study, we therefore hypothesized that rendering a feature dimension task relevant would strengthen IOG along this dimension. Demonstrating this effect would strengthen the case for functional similarities between typical binocular rivalry and IOG, and show that attentional modulation can impact percepts that combine inputs from two eyes (which has been demonstrated for the first time only recently, for stimuli very different from ours; see Acquafredda et al., 2022).

In our experiment, we used stimuli that contained two featural dimensions, and, depending on their variant, could elicit IOG percepts driven by each dimension individually or by both. In the former case, non-IOG percepts were driven by the other dimension and by the monocular information (i.e., the information about the eye to which certain input is presented, also called the eye-of-origin information). In the latter case, the non-IOG percepts were driven by the monocular information only. These stimuli were presented under two instructions, each designating a different dimension as task relevant and thereby applying attentional modulation to it. These dimensions were form (spatial coherence of a depicted object) and motion (motion direction). We used these to maximize the sensitivity of our experiment to task-related effects. Although form and motion processing begins in V1, which is heavily involved in the processing of monocular information, its core stages take place (relatively) late along the brain’s visual processing pipeline (Felleman & Van Essen, 1991; Van Essen & Gallant, 1994), and previous studies suggest that the proneness of typical binocular rivalry to task-related influences is higher in high-level rather than early vision (see Dieter & Tadin, 2011, for a review). Additionally, these features have been used in previous studies demonstrating the potential of attention to modulate typical binocular rivalry. For example, form stimuli were used by Chong et al. (2005; in their Experiment 2, they used complex static patterns) and by Van Ee et al. (2005), who relied on houses and faces, among other, less complex, stimuli. Motion stimuli, in turn, were used by Mitchell et al. (2004; rotating clouds of dots) and in multiple studies on the modulation of rivalry by input from other senses – for example, Lunghi et al. (2014) used dynamic noise-patterns, Blake et al. (2004) used random-dot cinematograms, and Liaw et al. (2022) used drifting gratings.

**Fig. 1:**
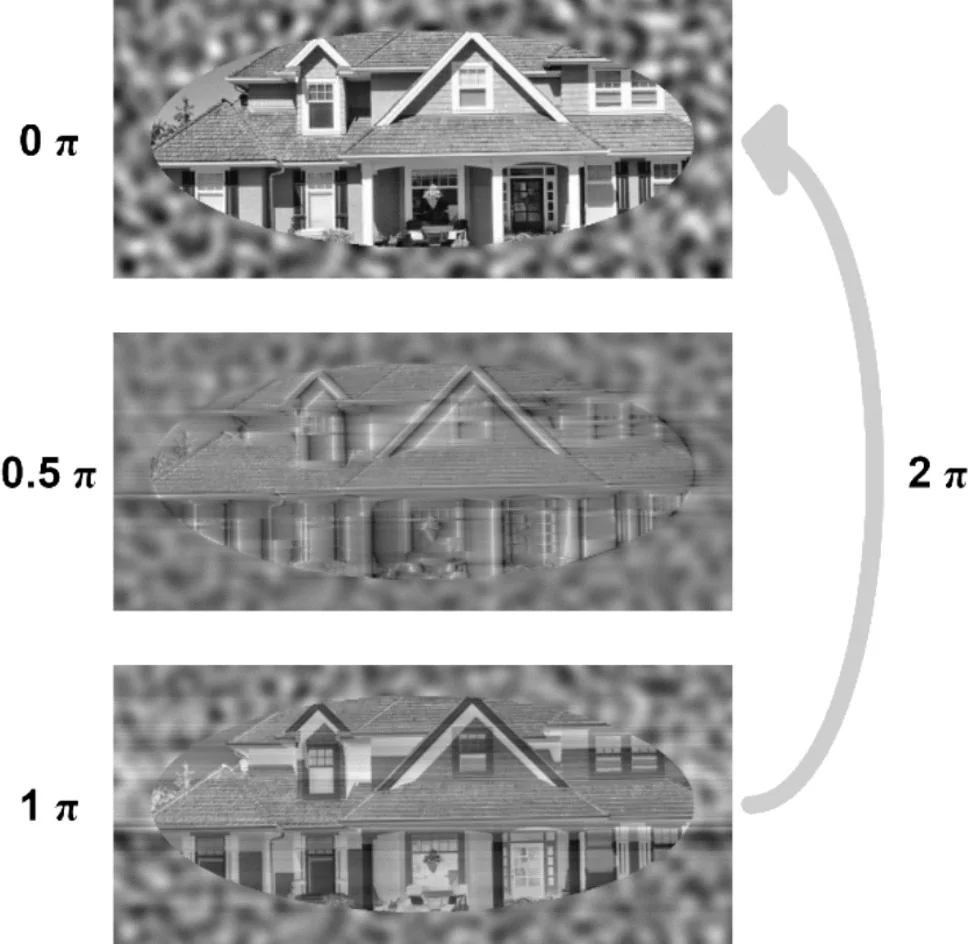
Effects of the phase-shift transform. Images from top to bottom are frames of the animations created by applying the phase shifts of, respectively, zero π, half π, and one π to an image. Note that the shift by half of the period (one π; last image) resulted in luminance polarity inversion. Shift by two π would result in recreating the first image. All three images shown here were extracted directly from stimuli presented in our study. These stimuli were created by concatenating multiple series (cycles) of 43 phase-shift transformed frames. Because the first and last frames were identical in each cycle, the concatenation thus allowed for creating animations moving continuously for a desired time. Images presented here are, respectively, the first, 12^th^, and 22nd (middle) frames of a single cycle. An animation demonstrating the effects of the phase-shift transform is available in the Online Supplementary Material.

To create our stimuli, we used a phase-shift image-transformation that allows presenting a static object as moving without changing its spatial location (Hayashi & Tanifuji, 2012; see also Freeman et al., 1991). This effect is achieved by dynamically changing luminance of different image regions in such a way as to induce perceived motion without distorting other visual features of the original (see Fig. 1). We subjected pairs of images of different objects to this transformation to obtain pairs of animations containing motion in opposite directions. By splitting each animation into halves along the vertical meridian and presenting different combinations of these halves to different eyes (similar to Diaz-Caneja’s demonstration), we independently manipulated form (i.e., object coherence) and motion (i.e., the direction of motion) presented to each hemifield of each eye. Depending on the pairing of stimulus halves, the inputs could give rise to IOG by form, by motion, or by form and motion, or they could elicit typical binocular rivalry. Importantly, we manipulated the task relevance of these two feature dimensions by instructing observers to report changes in either perceived motion direction or perceived form.

Using this experimental design, we aimed to assess whether IOG is influenced by task relevance, similar to typical binocular rivalry. In particular, we expected to observe more IOG along the task-relevant feature dimension. Moreover, based on previous studies (Holten et al., 2016; Papathomas et al., 2004; Stuit et al., 2011; Van Lier & De Weert, 2003; Vergeer & Van Lier, 2010), we expected stronger IOG when grouping was based on two, compared to one, feature dimension. We assessed IOG in two ways: first, we recorded the button presses by means of which observers were reporting changes in perception. Second, made possible by the use of motion direction as one of the featural dimensions along which IOG could occur, we used the traces of observers’ eye movements as an additional index for these changes (as is sometimes used in binocular rivalry studies with dynamic stimuli; Tsuchiya et al., 2015). Contrary to our predictions, we found that IOG was not strengthened by task relevance. Moreover, in contrast to previous reports, we found that IOG is not necessarily stronger with an increasing number of features driving grouping. However, our results also showed that under different tasks, observers moved their eyes differently, which affected the dynamics of changes in perceptual content they reported. Overall, our study suggests a less straightforward link between binocular rivalry and IOG than previously assumed.

## Method

### Stimuli

#### Images used in the experiment

We used two pairs of photographs of houses, acquired from the www.shutterstock.com webpage. We paired images in such a way that the depicted houses were clearly dissimilar. The photographs were converted to grayscale before further processing. This operation, as well as all other image-processing operations described here, were conducted in Matlab.

In each image, we masked regions close to the boundaries with a visual-noise pattern. The central region that was not masked had the shape of an ellipse, with a vertical axis 3.59 degrees of visual angle (dva) long and a horizontal axis with a length of 8.97 dva. The noise patterns surrounding the ellipse were generated for each image individually by bandpass filtering white visual noise with a 64th-order finite impulse response (FIR) filter with the passband between 0.03 *π* and 0.04 *π* radians per sample and then normalizing the resulting noise pattern to the [0, 1] range.

Before adding the noise patterns, the images were resized (but their aspect ratio was preserved) and cropped whenever these operations were necessary to align the depicted houses with the central region not masked by noise. Within each pair, these regions were matched in mean luminance and contrast using the SHINE toolbox (Willenbockel et al., 2010).

#### Phase-shift transform

After adding the noise ellipse, we converted each image into an animation in which the depicted the object is perceived to move without actually changing its position (for an animation showing this effect, see the Online Supplementary Material). This effect was achieved by using a phase-shift transform that is based on a two-dimensional Fourier transform (Hayashi & Tanifuji, 2012; see also Freeman et al., 1991, where a similar technique of displaying images is called “motion without movement”). In each frame of the animation, all spatial frequency components of a given frame have their phase incremented (“shifted”) by a fixed amount with respect to the previous frame (see Fig. 1). These operations generate systematic changes in the local luminance and give rise to a percept of motion in one direction. We used the phase-shift transform to convert each image into two animations: one with a leftwards (positive phase-shift) motion and one with a rightwards (negative phase shift) motion. The increment of phase between each of two consecutive frames was set to 1/21 π. This value was chosen based on pilot experiments.

Given that a phase shift of one full period (2 π) resulted in recreating the original image, animation frames with phase shifts ranging from 0 to 2 π constituted a single “cycle” (comprising 43 frames) in which the first and last frames were identical. Presenting many such cycles in a row (each with its last frame removed to avoid showing an identical frame twice in a row) allowed for presenting a continuously moving stimulus for a desired time interval.

#### Stimulus presentation

**Fig. 2:**
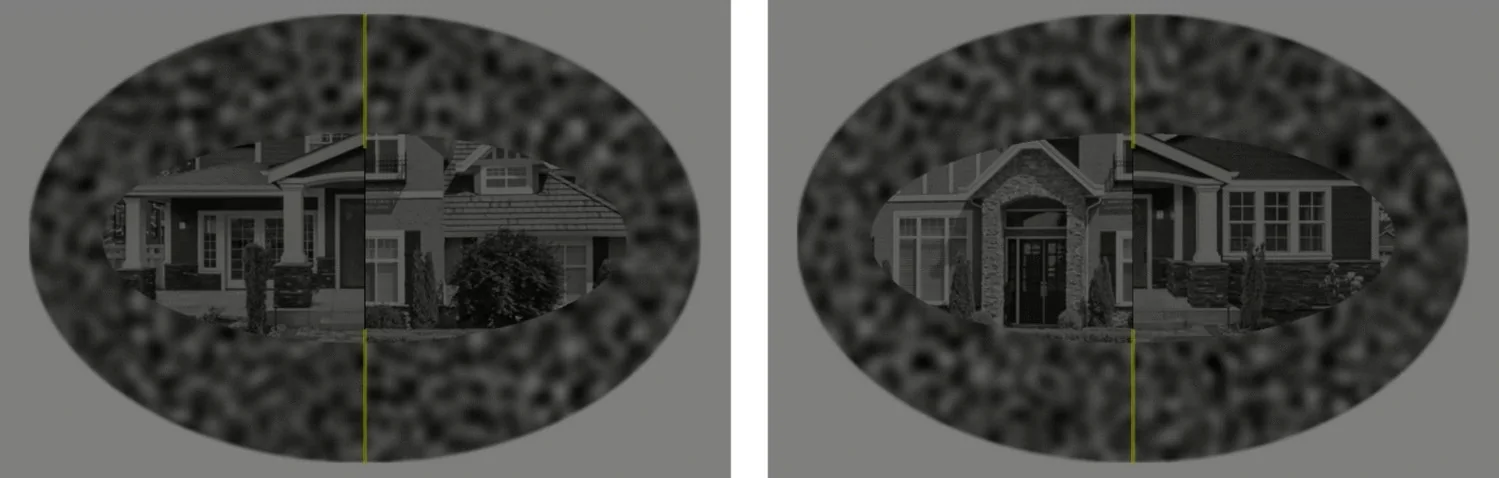
A single frame of stimuli shown to each eye. Note that (**1**) noise masks surrounding photographs were rivalling as well, and (**2**) horizontal bars placed above and below the stimuli to facilitate binocular fusion are now shown here.

Figure 2 shows a single animation frame as it was presented to one eye of an observer. Pixel intensity in all animations was reduced by multiplying all pixel values by 0.4 to avoid crosstalk between animations shown to different eyes (the stimuli were presented via shutter glasses; see *Apparatus* for further technical details). Visual inspection by two experienced experimenters indicated that there was no crosstalk in our setup. Each animation was presented through an ellipse-shaped aperture (axis lengths: vertical 7.67 dva, horizontal 11.5 dva) placed centrally on a screen against a gray background (see Fig. 1). The outer edges of the aperture were smoothed with a two-dimensional isotropic Gaussian kernel using the Matlab function imgaussfilt with the sigma set to two. The aperture was split into two halves by a vertical, one-pixel wide black line overlapping with a yellow-shaded (RGB values: 178, 178, 0) semi-transparent line having a width of around 0.06 dva (five pixels). The latter line was included to indicate the location towards which the observers should direct their gaze (i.e., the center of the screen). It had a 3.08 dva break in the middle. This break facilitated looking at the stimuli instead of strongly focusing on the line. On each trial, in each half of the aperture, different halves of the animation were presented to each eye (see Table 1). Above and below the aperture, identical horizontal bars containing binarized visual noise were shown to both eyes in order to facilitate binocular fusion and thus reliably elicit binocular rivalry (Law et al., 2013). Each bar had a width of 13.8 dva, a height of 1.84, and was located 0.77 dva above or below the ellipse. In the lower corners of a screen, we presented prompts indicating current trial instruction – see *Procedure* for details.

#### Design

Our sample was divided into two groups, with each group viewing animations derived from different image pairs. Each observer completed the experimental procedure in a single testing session. On each trial of the experiment, different halves of the animations were presented to the different hemifields of each eye. The combinations of the halves of animations had four variants that were defined by the featural dimension(s) that drove IOG (later called grouping cues, after Stuit et al., 2011): Motion (M), Form(F), Form and Motion (FM), and Null (N, for which IOG was impossible); see Table 1 for details.

In trials with each grouping cue, we counterbalanced both the identities of houses and the directions of motion in the animations. This counterbalancing resulted in four trials per cue. For example, for the Null grouping cue (which elicited typical binocular rivalry), in these four trials, the following stimuli were presented to the left eye: house A moving to the right (while house B moving to the *left* was presented to the right eye), house A moving to the left (while house B moving to the *right* was presented to the right eye), house B moving to the right, and house B moving to the left. See the Appendix for details of the counterbalancing schema we employed.

The four grouping cues and four unique trials per cue allowed for creating 16 unique pairs of stimuli. Each pair was presented twice (so the experiment consisted of 32 trials), each time with a different instruction given to observers. Under the Same Motion instruction, observers were asked to keep the “M” button pressed whenever perceiving motion in the same direction in both halves of the visual field and the “Z” whenever perceiving motion in opposite directions in the two halves. The Same Form instruction was similar, but instead of being ask to report the perceived motion, observers were asked to report whether they perceived halves of the same or different houses in the two halves of the visual field. Our task was therefore similar to the one used by Holten and colleagues (2016), in which observers continuously compared patterns perceived in two apertures. The relationship between the responses, grouping cues, and experiencing IOG percepts is presented in Table 1.

In each row, a combination of halves of animations corresponding to a different set of featural dimensions driving IOG (called the grouping cue) is presented, along with the instruction given to the observers. Capital letters A and B always indicate halves of the images AA and BB from which the animations used as stimuli were derived. An arrow above each letter indicates the direction of motion of the corresponding half of an animation. For example, the stimuli configuration listed in the second row can give rise to two possible outcomes: binocular rivalry (when the $\overrightarrow{A} \overleftarrow{A}$ percept is perceived intermittently with $\overleftarrow{B} \overrightarrow{B}$) and IOG by motion direction (when $\overrightarrow{A} \overrightarrow{B}$ is perceived intermittently with $\overleftarrow{B} \overleftarrow{A}$). The last row, in turn, illustrates that the grouping cue Null occurs when to each eye, a single, undivided image whose both halves move in the same direction is presented. For this cue, the IOG cannot occur along any dimensions (hence its name), so the typical binocular rivalry between $\overrightarrow{A} \overrightarrow{A}$ and $\overleftarrow{B} \overleftarrow{B}$ percepts is the only possible perceptual outcome for it. In different trials, depending on the combination of instruction and grouping cue (see the second and third columns), different button-press reports indicated that observers experienced IOG (fourth column). For example, for the grouping cue Form, under the Same Form instruction, observers could perceive the same form in both hemifields only as a consequence of IOG, so reporting “the same” indicated experiencing IOG percepts (see the very first row of the table, last column). Recall that for the grouping cue Null, IOG bias could not be calculated. Note that the illustration provided in the table does not take any counterbalancing into account

### Procedure

A testing session began with an experimenter verbally instructing the observer about the task and showing them animations eliciting the percepts they would need to report. Observers were also informed that the task was challenging, and they were asked to try to keep pressing one or the other button constantly throughout each trial. To familiarize themselves with the experience of rivalry, observers completed a 50-s training session resembling a single experimental trial. The stimuli used for training and instructions were always different from the ones used in the actual experiment.

The actual experiment comprised 32 trials presented in four blocks interleaved with breaks. The order of trials was randomized for each observer. Each trial lasted 105 s and was preceded by a centrally presented fixation dot displayed for 2 s. The instruction specific to a given trial was always displayed before the trial began. Observers had to press the space bar to start a trial, so they could take as much time to read the instructions as they wished. Additionally, during the trial, prompts reminding observers of the trial instruction were displayed in the lower corners of the screen. Specifically, each prompt indicated the response that should be given using a button on the side of a keyboard corresponding to the screen corner in which it was displayed (so the prompt in the left corner corresponded to the “Z” button and in the right corner to “M”).

#### Observers

We recruited 18 observers, of whom one was excluded because they provided very few button-press reports. The remaining observers were, on average, 23.24 years old, 13 identified as women, the rest identified as men. All declared having normal or corrected-to-normal vision. We expected that such a sample would be sufficient to reveal the effects of interest, given that previous studies on IOG used samples that were either smaller or similar in size (Gayet et al., 2015; Holten et al., 2016; Stuit et al., 2014).

All observers provided written informed consent before participating. The study was approved by The Research Ethics Committee at the Institute of Psychology, Jagiellonian University, and adhered to the tenets of the Declaration of Helsinki (Holm, 2019).

### Apparatus

The experimental procedure was programmed in MATLAB R2016a (MathWorks, Natick, MA, USA) and relied on routines from the Psychophysics Toolbox Version 3 (Kleiner et al., 2007). Each testing session took place in a dark room. Stimuli were displayed on a screen of a Dell Alienware 17 laptop controlling the experiment (resolution: 1,920 × 1,080 pixels; monitor diagonal: 17 in.). Observers viewed them through Nvidia 3D Vision 2 shutter glasses synchronized with the laptop via its built-in infrared emitter. Observers sat 88 cm from the screen, with their heads stabilized in a chin rest. They provided responses by pressing buttons on a computer keyboard connected to the laptop. Throughout the procedure, we recorded the eye movements of the observers using a desktop-mounted EyeLink 1000 eye tracker (SR Research, Ottawa, Canada) operating at a sampling rate of 1,000 Hz. It was calibrated using a five-point procedure at the beginning of each block of trials.

We used the NVIDIA shutter glasses at a refresh rate of 40 Hz for all but two observers, who were tested in a different testing room (with the same computer and setup but different EyeLink device) and a refresh rate of 60 Hz. Consequently, for these two observers, a single trial lasted 70 s instead of 105 s, and the speed of perceived motion was different. In our analyses, we pooled data from all 17 observers. However, we also analyzed the data of only the 15 consistent observers and found a similar pattern of results to the one reported here. We presented low-luminance stimuli in a dark room. Visual function therefore relied on rod-based, scotopic vision, for which the critical flicker-fusion threshold (i.e., the minimal frequency at which flickering stimuli appear to move in a smooth, non-jittery way) is below 40 Hz (Cao et al., 2006), ensuring that observers experienced a smooth motion percept.

## Results

We conducted all data wrangling and generated plots in R (version 4.2.1; R Core Team, 2022), relying on packages from the tidyverse collection (Wickham et al., 2019). Statistical analyses were carried out in JASP 0.95.4 (JASP Team, 2025). Our data and code can be accessed via the following link: 10.5281/zenodo.18924437.

### Results for the button-press metric

**Fig. 3:**
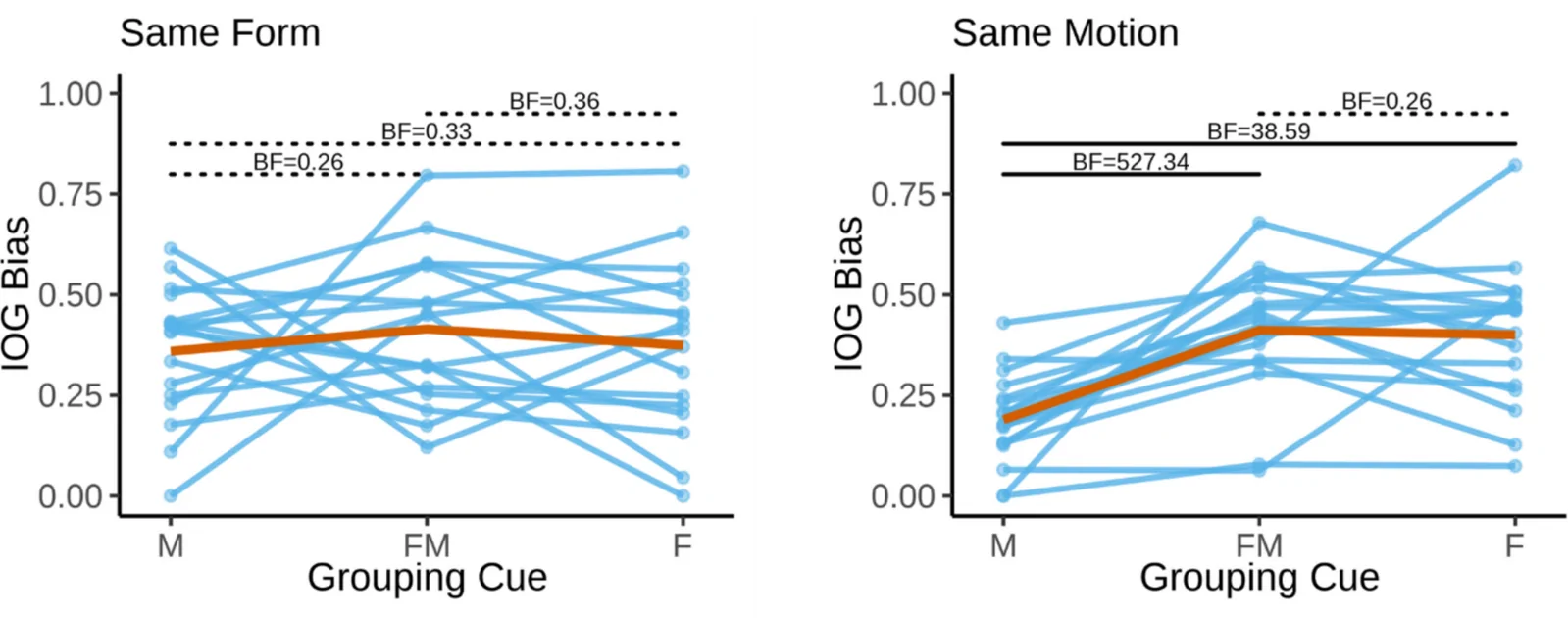
Interocular grouping (IOG) bias for ( Same Form and Same Motion instructions. In both panels, blue lines connect averages over trials of individual observers, while red lines connect the means of these averages. M = Motion, F = Form, FM – Form and Motion, BF = Bayes factor

To analyze the button presses, for each trial, we calculated the ratio between the total trial time when IOG percepts were reported (as defined in Table 1) and the trial time when any exclusive (i.e., indicated by pressing one of the buttons exclusively, not mixed) percepts were reported. This ratio served as a straightforward measure of bias towards IOG percepts (Stuit et al., 2014), with values ranging from 0 to 1. Values close to 1 indicate strong IOG, while values close to 0 indicate strong binocular rivalry, in which perceptual content is determined by monocular information. As illustrated in Fig. 3, for each observer, we averaged the obtained bias values over trials with the same grouping cues. In the trials with the grouping cue, Null IOG was impossible, so we excluded these trials from further analyses (but the plot showing these data is included in the Appendix). From the remaining trials, we excluded three trials in which observers reported mixed percepts for more than half of the trial duration. The analyses of IOG bias were thus conducted on data from 405 trials across 17 observers.

Before addressing our main question of interest, we wanted to confirm that all our stimuli elicited IOG. To this end, for each combination of instruction and grouping cue, we first calculated the average per-trial proportion of responses indicating IOG (i.e., responses included as the numerator in the calculation of the IOG bias) and responses indicating mixed percepts (which could result from genuine mixed percepts as well as “switching” between periods when different percepts were reported), and then compared them using paired-samples *t*-tests. In all cases, the proportion of responses indicating IOG was higher (all *p*-values below 0.001; see the full results in the Appendix), suggesting that these responses reflect genuine perceptual grouping.

The results for bias towards IOG percepts are shown in Fig. 3. They were analyzed using a Bayesian repeated-measures ANOVA with grouping cue and instruction as within-subject factors. We used the default prior (i.e., *r* = 0.5 for the fixed effects). To assess the robustness of the result, we also repeated the analysis for two different prior specifications (*r* = 0.2, *r* = 1). The interpretation of the Bayes factor was conducted in line with the guidelines provided by Lee and Wagenmakers (2014).

Table 2 shows Bayes factors for inclusion which was used to examine the effect of the grouping cue, instruction, and their interaction on IOG bias. It indicates that data were approximately 31 times more likely under models including the effect of grouping cue than under models not including this effect, approximately 19 times more likely under models including interaction of the grouping cue and instruction, and approximately five times more likely including the effect of instruction.

**Table 2:** Bayes factors (BFs) for inclusion characterizing how grouping cue and instruction jointly predict interocular grouping (IOG) bias

Effect	BF_inclusion_	BF interpretation
Grouping cue	31.31	Very strong evidence
Instruction	5.40	Moderate evidence
Grouping cue ✻ instruction	18.86	Strong evidence

We were primarily interested in examining whether designating a particular stimulus feature as task relevant increased bias towards IOG percepts driven by this feature. Therefore, we tested how this metric changed for each grouping cue between instructions with a Bayesian paired-samples I-test with a default JASP Cauchy prior (*r* = 0.707). We found moderate evidence for the lack of differences for the grouping cue F (Same Motion minus Same Form: *M*_diff_ = 0.03, BF = 0.28) and the cue FM (*M*_diff_ = 0.00, BF = 0.25). However, there was very strong evidence for the difference for M, but it had a direction opposite to what we expected: bias towards IOG percepts driven by motion was stronger under the Same Form instruction (*M*_diff_ = −0.17, BF = 61.01).

Next, we conducted a separate set of tests to examine how IOG bias differed between grouping cues within each instruction. Here, in line with what has been reported in the literature (Chopin & Mamassian, 2010; Einhäuser et al., 2021; Gayet et al., 2015), we expected to observe stronger grouping for FM than for the grouping cue currently not designated as task-relevant. We did not have specific predictions regarding the bias for FM and the task-relevant cue.

For both instruction types, our predictions were not confirmed. In trials in which observers received the Same Motion instruction, the bias for FM was similar to, rather than larger than, that for F (FM minus F: *M*_diff_ = 0.01, BF = 0.27, moderate evidence for null hypothesis) and stronger than that for M (FM minus M: *M*_diff_ = 0.22, BF = 527.34, extreme evidence for alternative hypothesis). Moreover, the bias for F was stronger than for M (F minus M: *M*_diff_ = 0.21, BF = 38.59, very strong evidence for alternative hypothesis). For the Same Form instruction, we found anecdotal or moderate evidence for lack of differences (FM minus F: *M*_diff_ = 0.04, BF = 0.36, anecdotal evidence for null hypothesis; FM minus M: *M*_diff_ = 0.05 BF=0.33, anecdotal evidence for null hypothesis; F minus M: *M*_diff_ = 0.01, BF = 0.25, moderate evidence for null hypothesis).

### Results for eye movements

#### Optokinetic nystagmus

Our stimuli provided an opportunity to examine a potential link between optokinetic nystagmus (OKN) and IOG. OKN is an involuntary oscillatory eye movement with a unique spatiotemporal signature: a slow phase, when the eyes follow the currently perceived motion, and a fast phase, when they are re-centered in a rapid, saccade-like shift (Distler & Hoffmann, 2011). It is triggered when unidirectional motion occupying a large part of the visual field is perceived, and is involuntary in the sense that it cannot be performed without the specific visual percept. Given its involuntary nature, and the fact that OKN reflects the contents of perception (at least typically, see Dakin & Turnbull, 2016; Veto et al., 2018), it is a well-established method of tracking perceptual alternations in binocular rivalry between stimuli moving in different directions (Aleshin et al., 2019; Einhäuser et al., 2017; Enoksson, 1964; Fox et al., 1975; Naber et al., 2011; Tsuchiya et al., 2015).

We explored the possibility of relating OKN traces to the observers’ perception as indexed by button-presses. We expected that in our experiment, all percepts of coherent, unidirectional motion (but not other percepts) would elicit OKN, irrespective of whether they resulted from IOG or not, which would have allowed us to analyze OKN traces analogously to button-presses. However, careful inspection of our data indicated no clear traces of OKN: the time courses of changes in horizontal eye position did not exhibit a characteristic sawtooth pattern, even for trials with a grouping cue of Null (i.e., the conventional binocular rivalry setup). This lack of OKN was not accompanied by excessive exclusion rates (reported in *Observers*, *Results for the button-press metric, *and in *Influence of instructions on eye movements*) or data loss (reported below). Therefore, although our data were of high quality overall, our stimuli were not eliciting OKN reliably, and we did not pursue analyses of OKN further.

#### Influence of instructions on eye movements

However, in an additional, exploratory analysis, we investigated whether eye movements in general were influenced by instructions. The dynamics of typical binocular rivalry is known to be tightly linked to eye movements (Naber et al., 2011; Van Dam & Van Ee, 2006a, b), and eye movements are closely related to motion signals. The additional analysis might therefore provide insights into the unexpected result that emerged under the Same Motion instruction, namely, *weaker* IOG for M compared to both FM and F cues.

We applied the following preprocessing to our eye-movements data: in each trial, we identified all samples for which eye position was not recorded (most likely due to blinks). We removed these series from the data, along with 100 samples leading and trailing them. Next, we calculated the percent of retained (valid) samples for each trial and excluded 40 trials in which this value was lower than 50%.

We assessed the influence of instructions on eye movements in an exploratory analysis, focusing on how the instruction variant and grouping cue impacted the variability of gaze position along the horizontal axis. We used this metric because it captures a range of relevant oculomotor behaviors such as ocular following elicited by stimulus motion or saccades to shift gaze between the right and left halves of the field of view. To measure that variability, for each trial, we calculated the interquartile range of eye position along the horizontal axis. Prior to analyzing those data, based on visual data inspection, we excluded as outliers six trials in which horizontal variability was higher than five dva. Moreover, all trials that were excluded from the button-press analysis were excluded from the eye-movements analysis too. In the retained 374 trials, the average fraction of eye-tracker samples in which the eye position was not recorded (data loss) amounted to 13.23% (*SD* = 13.25, median = 8).

To assess if horizontal variability of the gaze traces changed as a function of grouping cue and instructions type, we used grouping cue and instruction type as within-subject factors in a Bayesian repeated-measures ANOVA with the default prior. Table 3 shows Bayes factors for inclusion which was used to examine the effect of the grouping cue, instruction, and their interaction on horizontal variability.

**Table 3:** Bayes factors (BFs) for inclusion characterizing how grouping cue and instruction jointly predict horizontal variability in eye movements

Effect	BF_inclusion_	BF interpretation
Grouping cue	1.69	Anecdotal evidence
Instruction	6.80	Moderate evidence
Grouping cue ✻ instruction	3.80	Moderate evidence

**Fig. 4:**
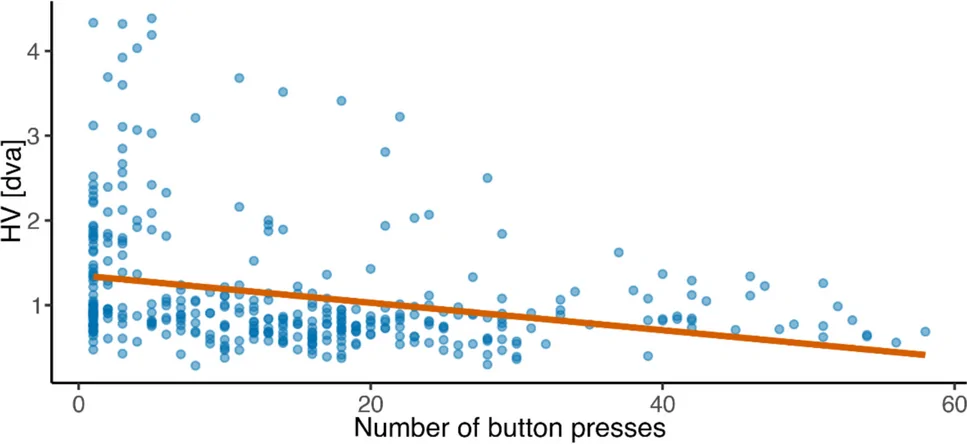
The relationship between the number of button-presses per trial and the horizontal variability in eye position (HV). Blue points indicate individual trials. The regression line plotted in orange was fitted to the data for visualization purposes.

Post hoc Bayesian paired *t*-tests indicated that horizontal variability was higher for the Same Form instruction (*M*_diff_ = −0.36, BF = 687.5, extreme evidence for alternative hypothesis). However, the pattern for the differences between the grouping cues was less straightforward. There was anecdotal evidence for the differences between FM and F (FM minus F: *M*_diff_ = 0.14, BF = 1.08) and anecdotal evidence for the lack of differences between FM and M condition (FM minus M: *M*_diff_ = 0.12, BF = 0.93). Given that the BF equal to one means no evidence for either the null or alternative hypothesis, the most plausible conclusion from the above-described pattern of results is that there is no conclusive information about effect or the lack of effect of the grouping cue on the horizontal variability. Still, there is moderate evidence that there are no differences between F and M conditions (F minus M: *M*_diff_ = −0.02, BF = 0.22). Overall, the data lend support to the hypothesis that instructions influenced eye movements: they were more spread out along the horizontal dimension under the Same Form instruction. This result, however, does not necessarily imply that the differences in gaze patterns were related to the changes in the rivalry dynamics. Although our data provided limited access to these dynamics (because observers were required to report only a subset of possible changes in perception), we still could index them using the total number of times in a trial when observers provided any of the two reports (pressed one of the two buttons). Therefore, to establish a link between the gaze behavior as captured by horizontal variability and the dynamics of perceptual fluctuations experienced by the observers, we calculated (Bayesian) the correlation between the variability values and the number of button presses (*r* = −0.28, BF = 324394, extreme evidence for the alternative hypothesis). Increasing horizontal variability values were associated with a decrease in the number of button presses, which suggests a link between the variability in gaze position and the dynamics of perceptual rivalry (see Fig. 4).

## Discussion

In this study, we investigated whether the strength of interocular grouping (IOG) is influenced by the number of featural dimensions driving the grouping and by the task relevance of these dimensions. While we find evidence for IOG in response to all cues, our results indicate that, in our specific setup, the number of cues does not increase IOG, which contrasts with previous reports. Furthermore, based on the ideas that (i) typical binocular rivalry and IOG are underpinned by the same neural machinery (Tong et al., 2006) and that (ii) in binocular rivalry, the task relevance of one of the rivaling stimuli increases its dominance (Chopin & Mamassian, 2010; Einhäuser et al., 2021; Gayet et al., 2015), we expected task relevance of a featural dimension to increase IOG along this dimension. Overall, we did not find this expected effect. While task relevance seemingly *weakened* IOG for one combination of instruction type and grouping cue, we show that this effect is potentially explained by the effect of task instructions on eye movements. Overall, in our specific setup neither the number nor the task relevance of grouping cues affects IOG per se.

The finding that the number of grouping cues had no influence on IOG strength was unexpected. It contrasts with previous reports (Holten et al., 2016; Stuit et al., 2011; Van Lier & De Weert, 2003; Vergeer & Van Lier, 2010), potentially indicating that not all feature dimensions are equally likely to be combined to increase grouping. This interpretation is partly supported by a previous study by Papathomas and colleagues (2004). While these authors showed that IOG driven by multiple features is stronger than IOG driven by a single feature, this effect was weaker when motion direction was one of the features involved. It is possible that both this result and the lack of additive effects of form and motion we observed stem from the fact that in the brain, form processing is dissociated from motion processing: form is processed primarily in the ventral visual stream, while motion is processed in the dorsal stream (Goodale & Milner, 1992; Mishkin et al., 1983). Combination of grouping features across the two streams might be particularly challenging in setups in which stationary features such as form would have to be combined with motion direction to drive IOG. Therefore, the extent to which an increase in IOG is seen as a consequence of an increased number of grouping cues might be dependent on the ease with which cues can be combined, with static cues being easier to combine with other static cues than with dynamics ones.

The lack of a strengthening effect of task relevance on IOG was our second unexpected finding. While the influence of task-related factors is underexplored in IOG (but see Acquafredda et al., 2022), their influence on typical binocular rivalry is well established (Chopin & Mamassian, 2010; Einhäuser et al., 2021; Gayet et al., 2015). Under the assumption that binocular rivalry and IOG are underpinned by (mostly) shared neural mechanisms, we therefore predicted to see more IOG along the task-relevant dimension. It seems unlikely that not finding this effect was due to observers misunderstanding the instructions: while the task itself was challenging for observers because of the fluctuating nature of the experienced percepts, the instruction manipulation was straightforward and simple. Rather, our results might indicate general differences in the role of task relevance between binocular rivalry and IOG which, in turn, could point towards differences in mechanisms underpinning these two phenomena. Alternatively, our findings might be specific to the feature dimensions and the task we used. For two reasons, we lean towards that view and are inclined to believe that our experiment would have yielded different results had the feature dimensions or the instructions been different. First, it has been found that the mutual influence of brain regions that process form and motion changes depending on the task (Jokisch & Jensen, 2007; Wokke et al., 2014). Second, our stimuli could engage magnocellular and parvocellular visual pathways, and each of these pathways handles interocular conflict differently (He et al., 2004). Therefore, the richness of our stimuli (especially the presence of motion in them in them) created ample potential for interactions within the visual system. While these interactions were too complex to pinpoint in our experiment, they could nevertheless be consistent with the idea that binocular rivalry and IOG rely on the same mechanisms. However, testing whether this consistency really holds would require additional data.

To our knowledge, there are no previous studies assessing the role of task relevance in IOG. However, our findings are in line with the few studies that have characterized the influence of other top-down factors. In particular, it has been found that even when IOG is possible, monocular information still remains a strong determinant of the contents of perception (see our Fig. 3) and that IOG strength is similar when stimulus parts that are interocularly grouped generate percepts that have a high-level interpretation (such as percepts of faces or optic flow patterns) and when they do not (Holten et al., 2016; Stuit et al., 2014; see also Lee & Blake, 2004; Mokri et al., 2023). The novelty of our study therefore lies in showing that this strengthening effect does not arise even when the high-level cues are designated as being task relevant. Interestingly, we chose these cues specifically because, according to the binocular rivalry literature (Dieter & Tadin, 2011), they should be more susceptible to task-related influences precisely because they are processed by lower- and, importantly, higher-level visual brain areas. Overall, however, our results suggest that IOG might be less prone to task influences than typical binocular rivalry.

While there was evidence that both grouping cues resulted in IOG in both instruction conditions, we also observed the surprising effect that the motion grouping cue resulted in *weaker* IOG under the Same Motion instruction as compared to the Same Form instruction. We relate this finding to the fact that under this instruction variability in horizontal eye position was also lower. The strategy likely adopted by observers under the Same Motion instruction was to track the motion direction. Somewhat paradoxically, this strategy resulted in less horizontally dispersed eye movements. This is because the images to which we applied the phase-shift transform contained multiple spatial frequencies. For such broadband spatial frequency images, the phase-shift transform generates a signal, for which there is no single motion velocity because each spatial frequency component has its own velocity (Hayashi & Tanifuji, 2012). If we assume that whenever an IOG percept of coherent motion was formed, observers started following with their eyes, then this situation creates a problem for the visual system: the percept does not contain a single velocity with which the eyes can move to stabilize the percept, which is analogous to how we employ eye movements to stabilize the retinal image of a moving surface under normal viewing conditions. Consequently, the displacement of frequency components that moved faster or slower than the eyes would remain uncompensated and might evoke changes in percepts akin to the ones elicited by retinal image slips. Such slips often cause switches in typical binocular rivalry (Van Dam & Van Ee, 2006a), and therefore it is likely that here, the uncompensated displacements kept disrupting the following ocular response and, consequently, IOG, which would explain why it was weaker under the Same Motion instruction. An additional analysis corroborated our conjecture about the link between eye movements and the changes in visual experience: the degree of the above-mentioned horizontal variability in gaze position was associated with one indicator of the dynamics of the perceptual rivalry, namely the number of times observers pressed buttons to provide a task response. Considered together, our results suggest that manipulating task instruction led to observers moving their eyes differently, which, in turn, impacted the dynamics of their perceptual experience.

In a previous study by Hayashi and Tanifuji (2012), phase-shift transformed images of faces induced optokinetic nystagmus (OKN), an involuntary eye movement that stabilizes the retinal image of a moving stimulus. These authors used OKN to track perceptual fluctuations in typical binocular rivalry between such stimuli, and, at least in principle, OKN might be similarly used with stimuli eliciting IOG. However, the analysis of our observers’ gaze traces did not contain any signature of OKN, even for stimuli giving rise to typical binocular rivalry. One possibility for the lack of OKN in our experiment is the reduction of stimulus contrast that was required to avoid crosstalk between monocular inputs. The phase-shift transform, which we used to induce motion percepts, relies on a dynamic modulation of stimulus contrast, and the contrast reduction likely weakened the motion signal. However, even low-contrast stimuli can elicit OKN reliably (Dakin & Turnbull, 2016; Leguire et al., 1991), and contrast reduction considered in isolation is unlikely to be the only reason for not observing the OKN in our experiment. Given that both stimulus velocity and spatial frequency spectrum impact OKN (Schor & Narayan, 1981; Waddington & Harris, 2015), additional factors at play could be the multitude of velocities with which the different spatial frequency components of stimuli were moving (discussed in the previous paragraph). In any case, our experiment warrants caution when using a phase-shift transformed image to elicit OKN. Another related challenge worth mentioning here is that popular algorithms for extracting perceived motion direction from OKN trace were developed for data obtained with stimuli in which motion velocity is constant (Aleshin et al., 2019), and, therefore, they might be unreliable when applied to data obtained using phase-shift transformed images.

To conclude, our study demonstrated that neither increasing the number of featural dimensions that can drive the IOG nor designating one such dimension as task relevant necessarily leads to stronger IOG. While these effects might be specific to the dimension we used (form and, especially, motion), our experiment highlights that IOG might be resistant to attentional modulation, either per se or because of the complex relationship between IOG and eye movements: our study demonstrated that IOG is sensitive to the observer's ongoing oculomotor behavior (which, in turn, depends on their current task). Revealing these complex interactions was possible because we created our stimuli using an innovative image-processing technique, namely the phase-shift transform (Hayashi & Tanifuji, 2012). Our study demonstrates the challenges associated with it and, therefore, can inform future experiments using this powerful yet not widely known method.

## Supplementary Information

Below is the link to the electronic supplementary material.

Supplementary file1 (DOCX 257 KB)

Supplementary file2 (MP4 259 KB)

## Data Availability

The data from this study can be accessed via the following link: 10.5281/zenodo.18924437

## Code availability

The code for running the analyses reported here can be accessed via the following link: 10.5281/zenodo.18924437
