# Supplementary material for: Neither the number of feature dimensions nor their task relevance necessarily affect interocular grouping in binocular rivalry
Source: Atten Percept Psychophys. 2026 May 6;88(5):125. doi: 10.3758/s13414-026-03265-0 (PMC13149590; doi:10.3758/s13414-026-03265-0)
Supplement: Supplementary file 1 — Supplementary file1 (DOCX 257 KB) [file 13414_2026_3265_MOESM1_ESM.docx]

**Neither the number of feature dimensions nor their task-relevance necessarily affect interocular grouping in binocular rivalry**

**Appendix**

Marek A. Pedziwiatr, Monika Derda, Weronika Bator, Michał Wierzchoń, Christoph Teufel

**1. Counterbalancing within a single testing session**

We explain the design and counterbalancing scheme we employed in our experiment based on Table A1, which contains a sample list of trials presented to a single observer. Each row of the table characterizes a single trial. The order of trials, unlike in the actual experiment, is not randomized. In this session, observer P1 (see Observer ID column) received the Same Form instruction in half of the trials, while in the other half – the Same Motion instruction (see Instruction column). The exact arrangements of animation halves presented to the different hemifields is specified in the Full Core column. The values in this column are strings of characters like D_1_R_x_D_2_R_xx_sIcore6 and contain (starting from its end):

1) the number indicating its ‘core’ (see Table A2)

2) information about counterbalancing (see below)

3) a separator _xx_

4) description of the stimulus presented to the left eye.

In this description, A_1_R means ‘left [coded as 1] half of the house A moving to the right [coded as R]’ etc. _x_ separates the specifications of animations presented to different hemifields. For example, A_1_R_x_A_2_R means ‘present whole house A moving to the right’. This information is sufficient to determine what stimulus was presented to the left eye. Letters C and D should be replaced with the letters A and B, respectively. We do not replace them here because we want the information in this Appendix to be consistent with our data, which is coded in that way for convenience reasons.

| Observer ID | Instruction | Full Core | Grouping Cue |
| --- | --- | --- | --- |
| P1 | Same Form | D_1_R_x_D_2_R_xx_sIcore6 | Null |
| P1 | Same Form | **D_1_L_x_D_2_R_xx_sIsMcore7** | M |
| P1 | Same Form | C_1_R_x_C_2_R_xx_core6 | Null |
| P1 | Same Form | C_1_R_x_D_2_L_xx_core5 | FM |
| P1 | Same Form | D_1_L_x_C_2_R_xx_sIsMcore5 | FM |
| P1 | Same Form | **C_1_L_x_C_2_R_xx_sMcore7** | M |
| P1 | Same Form | C_1_L_x_D_2_L_xx_sMcore8 | F |
| P1 | Same Form | **D_1_R_x_D_2_L_xx_sIcore7** | M |
| P1 | Same Form | D_1_L_x_D_2_L_xx_sIsMcore6 | Null |
| P1 | Same Form | C_1_L_x_D_2_R_xx_sMcore5 | FM |
| P1 | Same Form | C_1_R_x_D_2_R_xx_core8 | F |
| P1 | Same Form | D_1_R_x_C_2_R_xx_sIcore8 | F |
| P1 | Same Form | D_1_L_x_C_2_L_xx_sIsMcore8 | F |
| P1 | Same Form | C_1_L_x_C_2_L_xx_sMcore6 | Null |
| P1 | Same Form | **C_1_R_x_C_2_L_xx_core7** | M |
| P1 | Same Form | D_1_R_x_C_2_L_xx_sIcore5 | FM |
| P1 | Same Motion | A_1_R_x_A_2_L_xx_core3 | M |
| P1 | Same Motion | B_1_L_x_B_2_L_xx_sIsMcore2 | Null |
| P1 | Same Motion | A_1_L_x_A_2_R_xx_sMcore3 | M |
| P1 | Same Motion | B_1_R_x_B_2_R_xx_sIcore2 | Null |
| P1 | Same Motion | A_1_R_x_A_2_R_xx_core2 | Null |
| P1 | Same Motion | B_1_R_x_A_2_R_xx_sIcore4 | F |
| P1 | Same Motion | B_1_R_x_B_2_L_xx_sIcore3 | M |
| P1 | Same Motion | B_1_L_x_A_2_R_xx_sIsMcore1 | FM |
| P1 | Same Motion | A_1_L_x_B_2_L_xx_sMcore4 | F |
| P1 | Same Motion | B_1_L_x_A_2_L_xx_sIsMcore4 | F |
| P1 | Same Motion | A_1_L_x_B_2_R_xx_sMcore1 | FM |
| P1 | Same Motion | A_1_R_x_B_2_L_xx_core1 | FM |
| P1 | Same Motion | A_1_L_x_A_2_L_xx_sMcore2 | Null |
| P1 | Same Motion | B_1_L_x_B_2_R_xx_sIsMcore3 | M |
| P1 | Same Motion | B_1_R_x_A_2_L_xx_sIcore1 | FM |
| P1 | Same Motion | A_1_R_x_B_2_R_xx_core4 | F |

*Table A1. Specifications of trials from a sample testing session.*

The above-mentioned ‘core’ of a trial determined the instruction presented in it and a grouping cue at play (see also Grouping Cue column in Table A1). There were eight cores in total – see Table A2. From each core, we generated four trials (for counterbalancing; see below). Note that the grouping cues were identical for the following pairs of cores: 1 & 5, 2 & 6, 3 & 7, and 4 & 8.

| Core Number | Full Core | Grouping Cue | Instruction |
| --- | --- | --- | --- |
| 1 | A_1_R_x_B_2_L_xx_core1 | FM | Same Motion |
| 2 | A_1_R_x_A_2_R_xx_core2 | Null | Same Motion |
| 3 | A_1_R_x_A_2_L_xx_core3 | M | Same Motion |
| 4 | A_1_R_x_B_2_R_xx_core4 | F | Same Motion |
| 5 | C_1_R_x_D_2_L_xx_core5 | FM | Same Form |
| 6 | C_1_R_x_C_2_R_xx_core6 | Null | Same Form |
| 7 | C_1_R_x_C_2_L_xx_core7 | M | Same Form |
| 8 | C_1_R_x_D_2_R_xx_core8 | F | Same Form |

*Table A2. Cores defining trials (no counterbalancing is taken into account).*

To counterbalance motion directions and halves of animations derived from different photographs between the eyes, each core had four variants used in four different trials (see Full Core column in Table A2):

1. do not swap anything – a ‘basic’ version, listed in table A3 and changing in the remaining variants; it was not denoted by any letters.
2. swap motion directions – R became L and the other way round; denoted as sM
3. swap photographs – A became B or C becomes D; denoted as sI
4. swap both motion directions and photographs – the combination of the two above; denoted as sIsM

Each variant of each core was used only in a single trial. For illustration, consider all variants of core 7 in Table A1 (marked in bold in the Full Core column):

1. C_1_R_x_C_2_L_xx_core7
2. C_1_L_x_C_2_R_xx_**sM**core7
3. D_1_R_x_D_2_L_xx_**sI**core7
4. D_1_L_x_D_2_R_xx_**sIsM**core7

**2. Versions of Figure 3 from the main text with the grouping cue Null included**


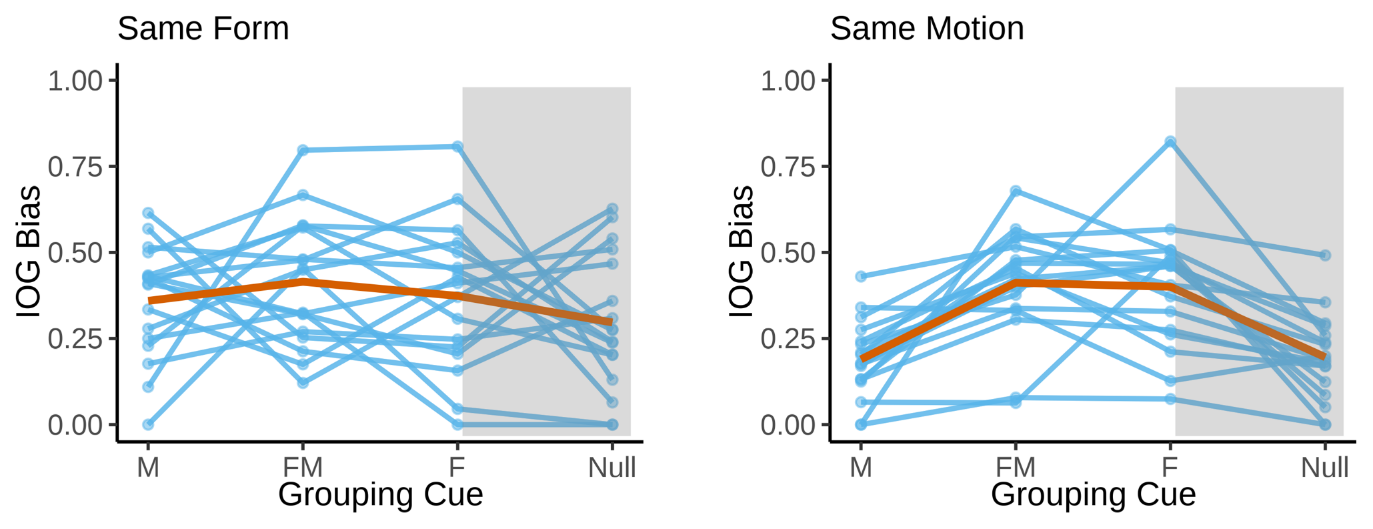


*Figure A1. Extended version of Figure 3 from the main text. The original figure shows IOG bias for the grouping cues M, FM, and F. This version additionally shows data for the grouping cue Null (against the grey background). Given that for this grouping cue, IOG bias could not be calculated (because in these trials, a typical binocular rivalry was taking place), instead of IOG bias, for this cue, we present the ratio of the total trial time when observers reported perceiving different contents in each hemifield to the trial time when they reported any exclusive (that is, not mixed) percept. Please refer to the caption of the original figure for data presentation details.*

**3. Additional results for section** **3.1 from the main text**

Paired-samples t-tests:

Motion, Same Object: M_diff_ = 0.28; t(66) = 8.69, p < .001

Form, Same Object: M_diff_ = 0.29; t(67) = 8.45, p < .001

Form and Motion, Same Object: M_diff_ = 0.33; t(66) = 10.38, p < .001

Motion, Same Motion: M_diff_ = 0.12; t(67) = 5.58, p < .001

Form, Same Motion: M_diff_ = 0.32; t(67) = 10.53, p < .001

Form and Motion, Same Motion: M_diff_ = 0.33; t(66) = 12.68, p < .001
